# Health-related quality of life in patients with severe COPD hospitalized for exacerbations - comparing EQ-5D, SF-12 and SGRQ

**DOI:** 10.1186/1477-7525-8-39

**Published:** 2010-04-15

**Authors:** Petra Menn, Norbert Weber, Rolf Holle

**Affiliations:** 1 Helmholtz Zentrum München - German Research Center for Environmental Health, Institute of Health Economics and Health Care Management, Neuherberg, Germany; 2 Asklepios Clinics München-Gauting, Gauting, Germany

## Abstract

**Background:**

The aim of this study was to measure HrQoL during acute exacerbations of COPD using generic and disease-specific instruments, and to assess completeness, proportion with best or worst health state, sensitivity to change and discriminative ability for each instrument.

**Methods:**

EQ-5D, SF-12 and SGRQ were obtained from COPD patients with GOLD stage III and IV hospitalized for an acute exacerbation both at admission and discharge. To assess the instruments' properties, utility values were calculated for EQ-5D and SF-12, and a total score was derived from the SGRQ.

**Results:**

Mean utilities ranged from 0.54 (SF-12, stage IV) to 0.62 (EQ-5D, stage III) at admission, and from 0.58 (SF-12, stage IV) to 0.84 (EQ-5D, stage III) at discharge. Completeness was best for EQ-5D and SGRQ, while no utility value for the SF-12 could be calculated for more than 30%. For SGRQ subscales, the minimal score occurred in up to 11% at admission, while full health was observed for the EQ-5D at discharge in 13%. Sensitivity to change was generally good, whereas discrimination between COPD stages was low for the EQ-5D.

**Conclusions:**

Acute exacerbations seriously impair health status and quality of life. The EQ-5D is generally suitable to measure HrQoL in exacerbations of severe COPD, although the high proportion of patients reporting full health at discharge poses a problem. The main issue with the SF-12 is the high proportion of missing values in a self-assessed setting. Properties of the SGRQ were satisfactory. However, since no utility values can be derived from this disease-specific instrument, it is not suitable for cost-utility analyses in health-economic evaluations.

## Background

Chronic obstructive pulmonary disease (COPD) is a common chronic condition that severely affects patients' health-related quality of life (HrQoL). With a prevalence of more than 13% in those aged 40 years and older in Germany, COPD is one of the most frequent causes of morbidity and mortality [1]. Exacerbations, acute worsenings of symptoms, have serious health consequences and are associated with an increased decline in lung function, hospitalization and even death [2]. It has been shown that on average, patients with severe or very severe COPD experience about 2.7 exacerbations per year, more than 10% of which require hospitalization [3].

A number of studies measure HrQoL in patients with COPD during stable phases of the disease using e.g. the generic EuroQol 5 dimension (EQ-5D) [4,5], the Short Form 12 (SF-12) [6], or the disease-specific St George's Respiratory Questionnaire (SGRQ) [4,7], but only few examine the effect of acute exacerbations. For stable disease, the EQ-5D's ability to discriminate between GOLD stages has been shown [4], but ceiling effects have also been reported [4,8]. However, properties of this instrument have not been assessed for acute exacerbations.

The most frequently used instruments to measure HrQoL during acute exacerbations are the SGRQ [9,10] and the Chronic Respiratory Disease Questionnaire (CRQ) [10,11]. As disease-specific instruments such as the SGRQ do not capture aspects of HrQoL unrelated to the specific disease and its consequences, utility values based on the SGRQ can not be compared with those based on generic instruments. To calculate quality-adjusted life-years (QALYs) for health-economic evaluations, only utilities based on generic instruments should be used. Utility values for exacerbated COPD based on generic instruments are essential when the cost-effectiveness of interventions is to be assessed that reduce the frequency or severity of exacerbations in COPD. To date, there is only one study that employs the EQ-5D for this question [12]. Yet it is not clear whether instruments such as the EQ-5D or the SF-12 are suitable to measure the impact of exacerbations on HrQoL.

Therefore, the aim of this study was to evaluate HrQoL using the two generic instruments EQ-5D and SF-12 and the disease-specific SGRQ in patients with severe and very severe COPD hospitalized for exacerbations, and to compare their results with regard to completeness, proportion with best and worst health state, sensitivity to change and discrimination between groups of different disease severity.

## Methods

### Study design

This prospective, observational study was conducted at the Asklepios Clinics in Gauting, Germany. Inclusion criteria were a minimum age of 45, a prior diagnosis of COPD and sufficient knowledge of the German language. All patients admitted between October 2007 and May 2008 for an exacerbation of COPD who met the inclusion criteria were asked to participate in the study. Participants signed a written consent form and answered a self-administered questionnaire within three days after admission and again within three days of discharge. Taking additional measurements during the hospital stay was not feasible for logistic reasons, nor was contacting patients after discharge for a follow-up. The questionnaire comprised the validated German versions of the EQ-5D, the SF-12 and the SGRQ to measure HrQoL as well as questions on patient's sex, age and smoking status. To assess disease severity, lung function was measured at discharge and categorized according to the GOLD classification [13]: Patients with a forced expiratory volume in 1 sec (FEV_1_) of 30-50% of their predicted value were categorized into stage III, patients with a FEV_1 _below 30% predicted, or with 30-50% predicted and chronic respiratory failure were categorized into stage IV. Patients with a FEV_1 _of more than 50% predicted were excluded. Patients who were readmitted to hospital during the study were asked to participate again.

### Instruments

#### EQ-5D

The EQ-5D is a generic questionnaire that consists of two parts: the descriptive section comprises the 5 dimensions mobility, self-care, usual activities, pain/discomfort and anxiety/depression [14]. Each dimension has 3 levels: no problems, some problems and severe problems. Respondents are asked to choose the appropriate level for each of the five dimensions. Utility scores can then be obtained by weighting the answers according to a weighting scheme. For base analysis, the German tariff was used to calculate utility scores [15]. Rutten-van Mölken et al. showed for the EQ-5D that utilities differ significantly depending on the value set used [4]. Thus, in addition to the German TTO weights, we also calculated utilities based on the UK tariff according to Dolan [16] to compare our results with studies from other countries.

The second part of the EQ-5D is a visual analogue scale (VAS). Respondents value their HrQoL on a rating scale from 0 (worst imaginable health state) to 100 (best imaginable health state).

#### SF-12

The SF-12 is also a generic instrument, containing 12 items selected from the SF-36. A physical and a mental component score (PCS and MCS) can be calculated. The PCS ranges from 11 to 60, the MCS from 16 to 70, with higher values indicating higher HrQoL. As no German value set was available for the SF-12, an international value set was used to obtain a preference-based health index (SF-6D) [17].

#### SGRQ

The SGRQ is a disease-specific instrument that consists of 50 items and was specifically developed for patients with chronic airflow limitation. Three component scores for the domains symptoms, activity and impact on daily life can be calculated as well as a total score [18]. Scores range from 100 to 0, with higher values indicating lower HrQoL. In contrast to the EQ-5D and the SF-12, no utility index can be obtained from this disease-specific instrument. For the analyses, the total score was used instead to assess the SGRQ's properties.

### Statistical analysis

Differences between baseline characteristics were tested using t-tests for continuous variables, and Pearson χ^2 ^tests for differences in percentages.

Completeness, proportion with best or worst health state, sensitivity to change and discrimination between the disease stages III and IV were considered to compare the instruments' applicability for COPD patients during acute exacerbations.

An instrument was considered complete if a utility or total score could be calculated. For this, in the EQ-5D, all 5 questions needed to be answered. For the SF-12, the items necessary for the calculation of the SF-6D were required. To calculate the SGRQ total score, the subscores symptoms, activity and impact had to be available. On each subscore, 2 to 6 missing items were allowed.

For each instrument, the proportion of patients with the best or worst possible health state was reported.

To assess sensitivity to change, standardized differences (sdiff) were calculated for the utility and total scores, respectively [19]. For this, absolute differences between mean values at discharge () and at admission () were divided by the standard deviation of admission values (*sd*_*A*_): . Paired t-tests were conducted to check significance on a 5% level. These analyses were repeated using the non-parametric Wilcoxon signed rank test for paired comparisons of clustered data as described by Rosner et al. [20] to account for the non-normality of the variables and the clustered data structure due to multiple hospital admissions of some patients.

The ability of an instrument to discriminate between disease stages was analyzed using linear mixed regression models, adjusting for age, sex, smoking status and time of assessment (admission or discharge). Random effects were employed to account for cluster effects due to multiple admissions. Analyses were rerun univariately using the non-parametric Wilcoxon rank test for clustered data [21] for those outcome variables where the assumption of a normal distribution was rejected, and conclusions were compared with those based on the parametric regression models.

## Results

### Patients

A total of 117 patients with GOLD stages III and IV participated in the study. Patient characteristics by disease stage are shown in Table 1. A total of 71% of patients had stage IV disease. Patients' age ranged from 45 to 88. Disease stages were comparable with respect to age, sex, smoking status and proportion with more than one admission, but differed significantly in length of stay, with longer stays for those in stage IV. In all, 8 patients were observed twice and 1 patient was observed three times. Therefore, the 117 patients represent 127 admissions.

**Table 1:** Patient characteristics by disease stage for first admissions

Characteristics	Stage III (n = 34)	Stage IV (n = 83)	p-value
age (mean (sd))	67 (8)	68 (8)	0.77*
male (%)	59	66	0.53^†^
current smoker (%)	18	14	0.78^†^
> 1 admission (%)	6	10	0.44^†^
length of stay [days] (mean (sd))	10 (6)	15 (8)	<0.001*

* t test;

^† ^χ^2 ^test.

### Health-related quality of life

Table 2 summarizes the distribution of the EQ-5D and SF-6D results. For the EQ-5D, the most frequent category at admission was 'some problems' on all dimensions. At discharge, this changed to 'no problems' for the dimensions self care and anxiety/depression. For the SF-6D dimensions physical functioning and role limitation, the worst category was most frequent at both time points, but improved from level 4 to level 3 for the dimensions social functioning and mental health from admission to discharge.

**Table 2:** Distribution of EQ-5D and SF-6D results (%)

EQ-5D	Level	Mobility	Self Care	Usual Activities	Pain/Discomfort	Anxiety/Depression	
Admission	1	8.9	32.5	7.3	19.0	36.6	
	2	**89.4**	**65.0**	**71.5**	**63.8**	**53.7**	
	3	1.6	2.5	21.1	17.2	9.8	
	n	123	120	123	116	123	

Discharge	1	37.3	**49.2**	21.4	41.9	**48.8**	
	2	**62.7**	47.6	**70.6**	**54.0**	48.0	
	3	0.0	3.2	7.9	4.0	3.2	
	n	126	126	126	124	125	

**SF-6D**	**Level**	**Physical funct.**	**Role lim.**	**Social funct.**	**Pain**	**Mental Health**	**Vitality**

Admission	1	3.3	1.9	7.4	15.3	10.8	1.0
	2	17.1	26.9	8.3	11.9	15.0	4.9
	3	**79.7**	2.9	34.7	17.8	30.0	1.9
	4	-	**68.3**	**37.2**	**40.7**	**35.0**	28.2
	5	-	-	12.4	14.4	6.7	**37.9**
	6	-	-	-	-	2.5	26.2
	n	123	104	121	118	120	103

Discharge	1	4.0	1.9	4.9	21.0	12.0	1.0
	2	44.4	29.0	18.9	13.5	17.1	3.0
	3	**51.6**	0.0	**35.3**	16.8	**39.3**	11.9
	4	-	**69.2**	33.6	**40.3**	23.9	27.7
	5	-	-	7.4	8.4	5.1	**41.6**
	6	-	-	-	-.	2.6	14.9
	n	124	107	122	119	117	101

Mean (sd) values for HrQoL at admission and discharge by disease severity are shown in Table 3. For all instruments, HrQoL improved from admission to discharge, and apart from the SGRQ symptoms score it was better in stage III than in stage IV. However, the size of this effect differed between the instruments: while for the EQ-5D, differences between disease stages were small, values increased considerably from admission to discharge. The SF-6D on the other hand improved only slightly with time, yet stage III patients showed consistently better HrQoL than stage IV patients. Correlations between EQ-5D and SF-6D were 0.43, between EQ-5D and SGRQ total -0.59, and between SF-6D and SGRQ total -0.57.

**Table 3:** Mean (sd) values for HrQoL at admission and discharge by disease severity

	Admission		Discharge	
	Stage III	Stage IV	Stage III	Stage IV
EQ-5D	0.62 (0.26)	0.60 (0.26)	0.84 (0.20)	0.75 (0.22)
VAS	42 (16)	37 (13)	63 (14)	52 (17)
SF-12 - SF-6D	0.61 (0.13)	0.54 (0.08)	0.65 (0.12)	0.58 (0.08)
SF-12 - PCS	28 (8)	27 (5)	34 (6)	31 (6)
SF-12 - MCS	47 (11)	39 (10)	47 (10)	41 (10)
SGRQ - total	67 (13)	72 (11)	58 (15)	66 (13)
SGRQ - symptoms	76 (17)	73 (16)	73 (18)	70 (17)
SGRQ - activity	82 (13)	87 (11)	73 (17)	84 (14)
SGRQ - impact	56 (17)	62 (14)	46 (19)	56 (18)

MCS: mental component score; PCS: physical component score.

For comparison with studies from other countries, an EQ-5D index based on the UK tariff according to Dolan [16] was calculated additionally. Mean (SD) values for the UK index of the EQ-5D at admission were 0.46 (0.31) in stage III and 0.44 (0.31) in stage IV, and at discharge 0.72 (0.23) and 0.61 (0.28), respectively. This difference between the German and the UK values is due to differing regression equations in calculating the utilities, where the German tariff assigns higher values to the same health states [15].

**Figure 1:**
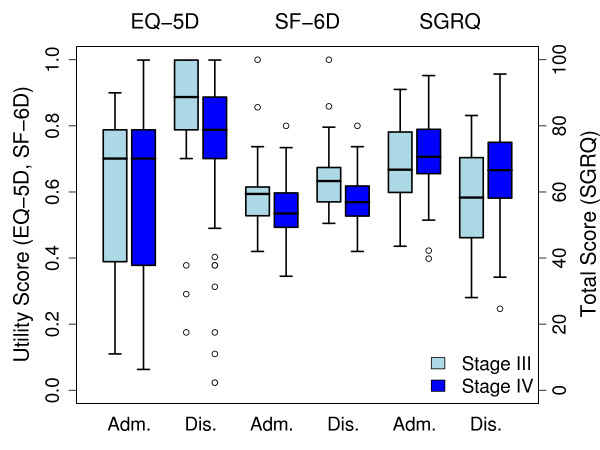
**HrQoL by disease severity and time of assessment for the 3 instruments**. Adm.: Admission; Dis.: Discharge.

Figure 1 shows boxplots of the utility and total scores, respectively, at admission and discharge by disease severity for the 3 instruments.

### Completeness

Table 4 (col. 2-4) shows the proportion of questionnaires for each instrument, where a calculation of a utility or total score was not possible due to missing items. Completeness was best for EQ-5D and SGRQ, whereas the SF-12 showed the highest proportion of missing values with more than 30% without a utility score. Utilities and total scores according to EQ-5D and SGRQ were slightly higher (not significant) for the subgroup with complete SF-12 questionnaires compared to those with missing SF-6D, but disease severity did not differ between patients with available and missing scores. In all instruments, missing items were more frequent in women than in men (11% vs. 6% for EQ-5D, 38% vs. 27% for SF-12, 12% vs. 4% for SGRQ), as well as in patients aged 68 and older (10% vs. 6% for EQ-5D, 41% vs. 21% for SF-12, 13% vs. 2% for SGRQ).

**Table 4:** Completeness and proportion with best and worst health state by instrument

	Missing utility/total score			Worst health state		Best health state	
Instrument	Adm.	Dis.	Total	Adm.	Dis.	Adm.	Dis.
EQ-5D	12%	3%	8%	-	-	2%	13%
SF-12	30%	32%	31%	2%	-	-	-
SGRQ - total	6%	8%	7%	-	-	-	-
SGRQ - impact				-	-	-	-
SGRQ - symptoms				5%	-	-	-
SGRQ - activity				11%	11%	-	-

Adm.: Admission; Dis.: Discharge.

### Proportion with best or worst health state

Table 4 (col. 5-8) summarizes the proportion of patients reporting best or worst health state at admission and discharge. While at admission, the worst state did not occur for the EQ-5D and the SF-12, it was observed for the SGRQ subscores symptoms and activity in 5% and 11% of patients, respectively. For the activity subscore, this remained unchanged at discharge. In the EQ-5D, 13% of all patients reported no problems in all 5 dimensions at discharge. These proportions were similar for both disease stages in the EQ-5D and the SGRQ symptoms score, but were more frequent for the SGRQ activity score in stage IV. Patients with full health according to the EQ-5D had SF-6D values from 0.55 to 0.86. All of them reported health restraints in the vitality dimension, 83% in mental health and 80% in physical functioning.

If instead of best health state, the proportion with maximum utility score was considered, this percentage rose from 13% to 23% for the EQ-5D at discharge. The reason for this is that the item 'usual activities' is not included in the weighting scheme of the German tariff (only in the form of a dummy variable indicating if 'extreme problems' were reported on any dimension). Maximum EQ-5D utility scores were more frequent in stage III than in stage IV (35% vs. 18%).

### Sensitivity to change

All instruments reported higher HrQoL at discharge compared to admission, with absolute standardized differences from 0.13 to 1.17 (Table 5), and except for the SF-12 MCS, differences were significant on the 5% level. The non-parametric Wilcoxon signed rank test for paired comparisons of clustered data yielded consistent results.

**Table 5:** Sensitivity to change

Instrument	n	Admission		Discharge		sdiff	p-value
		mean	(sd)	mean	(sd)		
EQ-5D	106	0.60	(0.26)	0.79	(0.21)	0.69	<0.001
VAS	120	39	(14)	55	(17)	1.17	<0.001
SF-12 - SF-6D	68	0.56	(0.11)	0.59	(0.09)	0.27	0.008
SF-12 - PCS	49	28	(6)	32	(6)	0.75	<0.001
SF-12 - MCS	49	41	(11)	42	(11)	0.13	0.308
SGRQ - total	111	71	(12)	65	(14)	0.50	<0.001
SGRQ - symptoms	118	75	(17)	71	(17)	0.23	<0.001
SGRQ - activity	116	86	(12)	81	(15)	0.37	<0.001
SGRQ - impact	116	61	(15)	53	(19)	0.48	<0.001

MCS: mental component score; PCS: physical component score; sdiff: standardized difference.

### Difference between disease stages

The results of the mixed linear regression models are shown in Table 6. Values are adjusted for sex, age, smoking status and time of assessment (admission or discharge). Interaction terms between time of assessment and disease stage were tested but were not significant. Apart from the SGRQ symptoms score, all instruments reported higher HrQoL in stage III than in stage IV. Except for the symptoms score, the EQ-5D and the SF-12 PCS, these differences were significant on the 5% level. With regard to the SF-6D, scores of patients in stage IV were particularly worse in the dimensions social functioning and role limitations. The non-parametric Wilcoxon rank test for clustered data yielded consistent results.

**Table 6:** Difference between disease stages

Instrument	n	Stage III	Stage IV	p-value*
EQ-5D	231	0.73	0.68	0.180
VAS	243	52	45	0.009
SF-12 - SF-6D	173	0.62	0.56	0.003
SF-12 - PCS	141	30	30	0.599
SF-12 - MCS	141	47	41	0.007
SGRQ - total	234	63	69	0.008
SGRQ - symptoms	243	75	72	0.317
SGRQ - activity	240	78	85	0.008
SGRQ - impact	240	51	59	0.005

* from mixed linear regression adjusted for age, sex, smoking status and time of assessment

MCS: mental component score; PCS: physical component score.

## Discussion

One disease-specific (SGRQ) and two generic (EQ-5D, SF-12) instruments were used to measure HrQoL at admission and discharge in patients with severe and very severe COPD hospitalized for acute exacerbations. Objectives of the study were to evaluate HrQoL during acute exacerbations and to compare the 3 instruments with regard to completeness, proportion with best or worst health state, sensitivity to change and discrimination between groups of different disease severities.

The main problem of the SF-12 in this self-administered setting was the high proportion of missing values. For less than 55% of all patients, a comparison of utility values at admission and at discharge was possible. Half of all missing utilities were due only to 2 items of the SF-6D. As one missing item precludes the calculation of a utility, and since the proportion of missing values increases with age, a high percentage of missing utilities was observed in this relatively old patient group. For the SGRQ on the other hand, subscores and a total score can still be calculated with up to 6 missing items per subscore. Therefore, completeness was best for the SGRQ in spite of its length of 50 items.

Worst possible scores were observed for the symptom score of the SGRQ at admission, as well as for the activity subscore at both time points. However, whereas this is tolerable for a disease-specific instrument in this severely ill patient group, the relatively high proportion with full health according to the EQ-5D at discharge poses a more serious problem. Ceiling effects were known to be present in stable phases of less severe COPD stages [4], but in our study the best possible state in EQ-5D was observed in severe and very severe COPD at discharge, while corresponding SF-6D scores were as low as 0.55 and patients reported health restraints in vitality (100%), mental health (83%) and physical functioning (80%). Therefore, the EQ-5D might not be sensitive enough to capture the health restraints that without doubt are still present in patients with severe COPD at discharge from a hospital-treated exacerbation. However, our results are in line with other studies. In a study on patients from various disease groups, full health in EQ-5D was observed in 9% of patients, of whom 92% reported health restraints in SF-6D dimension vitality, 65% in mental health, 71% in physical functioning [22]. And in a study on liver transplant patients, full health in EQ-5D was observed in 16%, of whom 94% reported health restraints in vitality, 51% in mental health, 80% in physical functioning [23]. SF-6D scores in individuals with full health in EQ-5D ranged as low as 0.57 and 0.56, respectively.

Sensitivity to change was generally good in all instruments. However, while the SF-12 PCS improved significantly from admission to discharge, the MCS showed only marginal changes. The reason for this might be that it requires more time for patients' mental state to recover from an exacerbation than it does for their physical condition. Differences between the instruments may be due to different reporting periods: the EQ-5D asks for the patient's immediate situation ("today"), whereas the reporting periods of the SF-12 and the SGRQ were 4 weeks and 3 months, respectively. In exacerbations, which usually show an acute onset of health status deterioration, a recall time of 4 weeks or more may be too long to detect these rapid changes. On the other hand, it is not clear how much patients pay attention to the respective reporting periods, particularly when answering the 3 instruments consecutively.

Differences between disease stages were observed for the MCS, but not for the PCS. While these differences are known to be present in stable phases of the disease [6], they may be reduced in the physical dimension during acute exacerbations.

As previous studies [8,24], we found a higher variance in utility values derived from the EQ-5D compared to the SF-6D. Also, mean EQ-5D utilities were higher than SF-6D utilities. Grieve et al. name the absence of a vitality dimension in the EQ-5D as a possible explanation [24]. In our study, more than 90% of all patients reported in the SF-12 at admission that they had a lot of energy 'some of the time' or less, at discharge, this still held for more than 80%. The effect of this aspect of HrQoL may not fully be captured by the EQ-5D, which may result in higher utilities.

EQ-5D utilities based on the UK tariff and VAS values at admission in our study were considerably higher compared to those by O'Reilly et al. [12], and still somewhat higher at discharge. One reason for this might be that those patients who were most impaired could not take part in the study, because they were not able to complete the questionnaire. As we applied 3 instruments instead of the EQ-5D only, our questionnaire was considerably longer, which may have caused more patients to deny participation. This probably underestimates the health impairment by exacerbations. Yet for patients participating in the study, no association between the presence of missing utility values and disease severity was observed. Also, although the time frame for assessing the questionnaire was within 3 days of admittance in both studies, there may have been differences in average time. In our study, about 30% of patients completed the questionnaire on the day of admittance, and about another 50% on the day after. O'Reilly et al. do not specify this issue, but if the majority of patients completed their questionnaire on the day of admittance, this may explain some of the differences observed. Differences in HrQoL might also be due to differences in the countries' health care systems. If patients are admitted earlier in Germany than they are in the UK, this could result in better HrQoL. Another reason might be that our staging was based on lung function at discharge, while O'Reilly used the last recorded FEV_1 _in General Practitioner notes which might result in less severe COPD stages. However, HrQoL at discharge as measured by the EQ-5D (UK tariff) and the VAS in our study were only slightly below those observed by Rutten-van Mölken et al. in stable stage III and IV [4], as were mean values for SF-12 PCS and MCS at discharge compared with Garrido et al. [6], which might indicate appropriate staging. However, HrQoL at discharge according to all SGRQ scores was still considerably worse than in stable disease phases [4].

Doll et al. found SGRQ scores of 40 to 80 during exacerbations [9]. The results of the present study agree with these findings with values between 50 and 80 at discharge. Higher values were observed at admission, ranging from 60 to 90. For patients with chronic bronchitis, Doll et al. found a decrease by 7, 2, 7 and 8 points for the SGRQ total, symptoms, activity and impact score, respectively, from exacerbation to stable phase [9]. In our study, this reduction was similar with 6, 4, 5 and 8 points.

One limitation of our study is that missing values were more frequent in women and in older patients. Especially in the SF-12, this probably resulted in an overestimation of HrQoL, whereas the proportion with worst health state may be underestimated. Sensitivity to change might also be affected, if those more likely to be missing are also more likely to improve from admission to discharge. Furthermore, the most severely ill patients could not be included in the study since they were not able to answer the questionnaire. This is most likely to lead to an overestimation of HrQoL in our study, but the amount of this bias is not known. Also, the time course of patients' health state after discharge was not observed within this study. This information is useful to calculate QALY loss associated with severe exacerbations more precisely. Results from O'Reilly in a subgroup of patients indicate that utility values had dropped 3 months after discharge. However, further research is needed to confirm these findings for other instruments such as the SF-12.

Also, no information on patients' comorbidities was available. However, Rutten-van Mölken et al. found no significant differences in the number of concomitant diagnoses or the Charlson comorbidity index between GOLD stages [4], so the differences in HrQoL between disease stages that were observed can be expected to persist after an adjustment for comorbidity.

In all, this study showed that generic instruments as the EQ-5D or the SF-12 are suitable to measure HrQoL during acute exacerbations and show good properties for most criteria.

## Conclusions

Acute exacerbations have serious effects on health status and quality of life.

In all, the EQ-5D appears to be suitable to measure HrQoL in this patient group, although the relatively high proportion with full health poses a problem. Still, completeness was at 92%, sensitivity to change was satisfactory, and this instrument is easy to apply due to its brevity. The SF-12 appears less suitable for a self-assessed setting due to the high proportion of missing values, although complete questionnaires showed good properties in the remaining aspects. Properties of the SGRQ were generally good. However, no utility values can be derived for health-economic evaluations from this disease-specific instrument.

## Competing interests

The authors declare that they have no competing interests. This work was supported by the "Kompetenznetz Asthma/COPD (Competence Network Asthma/COPD)" funded by the Federal Ministry of Education and Research (FKZ 01GI0881-0888).

## Authors' contributions

PM participated in the design and the coordination of the study, performed the statistical analysis and wrote the manuscript. NW participated in the design and the coordination of the study. RH supervised the study and assisted in writing the manuscript. All authors read and approved the final manuscript.
